# High resolution melting analysis: rapid and precise characterisation of recombinant influenza A genomes

**DOI:** 10.1186/1743-422X-10-284

**Published:** 2013-09-12

**Authors:** Donata Kalthoff, Martin Beer, Bernd Hoffmann

**Affiliations:** 1 Institute of Diagnostic Virology, Friedrich-Loeffler-Institut, Südufer 10, Greifswald-Insel Riems, 17493, Germany

**Keywords:** Influenza a virus, Highly pathogenic avian influenza virus, High resolution melting analysis

## Abstract

**Background:**

High resolution melting analysis (HRM) is a rapid and cost-effective technique for the characterisation of PCR amplicons. Because the reverse genetics of segmented influenza A viruses allows the generation of numerous influenza A virus reassortants within a short time, methods for the rapid selection of the correct recombinants are very useful.

**Methods:**

PCR primer pairs covering the single nucleotide polymorphism (SNP) positions of two different influenza A H5N1 strains were designed. Reassortants of the two different H5N1 isolates were used as a model to prove the suitability of HRM for the selection of the correct recombinants. Furthermore, two different cycler instruments were compared.

**Results:**

Both cycler instruments generated comparable average melting peaks, which allowed the easy identification and selection of the correct cloned segments or reassorted viruses.

**Conclusions:**

HRM is a highly suitable method for the rapid and precise characterisation of cloned influenza A genomes.

## Background

Influenza A viruses (IAV) are pathogens of major importance in both public health and veterinary medicine and have a high socio-economic impact. Research has generated a substantial increase in our knowledge of influenza viruses in recent years. A technique that has revolutionised influenza research is plasmid-based reverse genetics, which enables the generation of custom-designed recombinant viruses [1,2]. Routine cloning procedures are the standard for manipulating the viral genome and thus facilitate basic and applied research. The comparative segment exchange between different influenza virus strains with plasmid-based sequences and the subsequent generation of designed influenza virus reassortants is a major research technique because it facilitates studies on many topics, such as the molecular basis of IAV pathogenesis.

To date, the sequencing of cloned influenza segment plasmids and generated reassortant viruses has been necessary to confirm the successful implementation of sequence mutations. However, novel versatile technologies for the rapid and precise detection of mutations or single nucleotide polymorphisms (SNP) are currently available. In this study, we describe the application of “High-Resolution Melt (HRM) Analysis” for the verification of newly generated IAV reassortants (with mutations in a single segment) as an exemplary practical approach.

The principle of HRM analysis is based on the melting (dissociation) behaviour of DNA as it changes its transition from double- to single-strand status in the presence of a saturating fluorescent DNA-binding dye [3]. Melting analysis detects differences in the PCR amplicons that depend on the length, base composition, and strand base pairing of the amplicon. Genotyping, microbial detection, and species identification are the main fields in current HRM analysis [4-8]. Diagnostic strategies using HRM analysis have also been developed for influenza A subtyping and the detection of resistance to neuraminidase inhibitors from human samples [9-13].

Our study focused on the identification of influenza A viruses that were generated *in vitro* using a reverse genetics technology [14] through HRM analysis.

## Results and discussion

**Figure 1:**
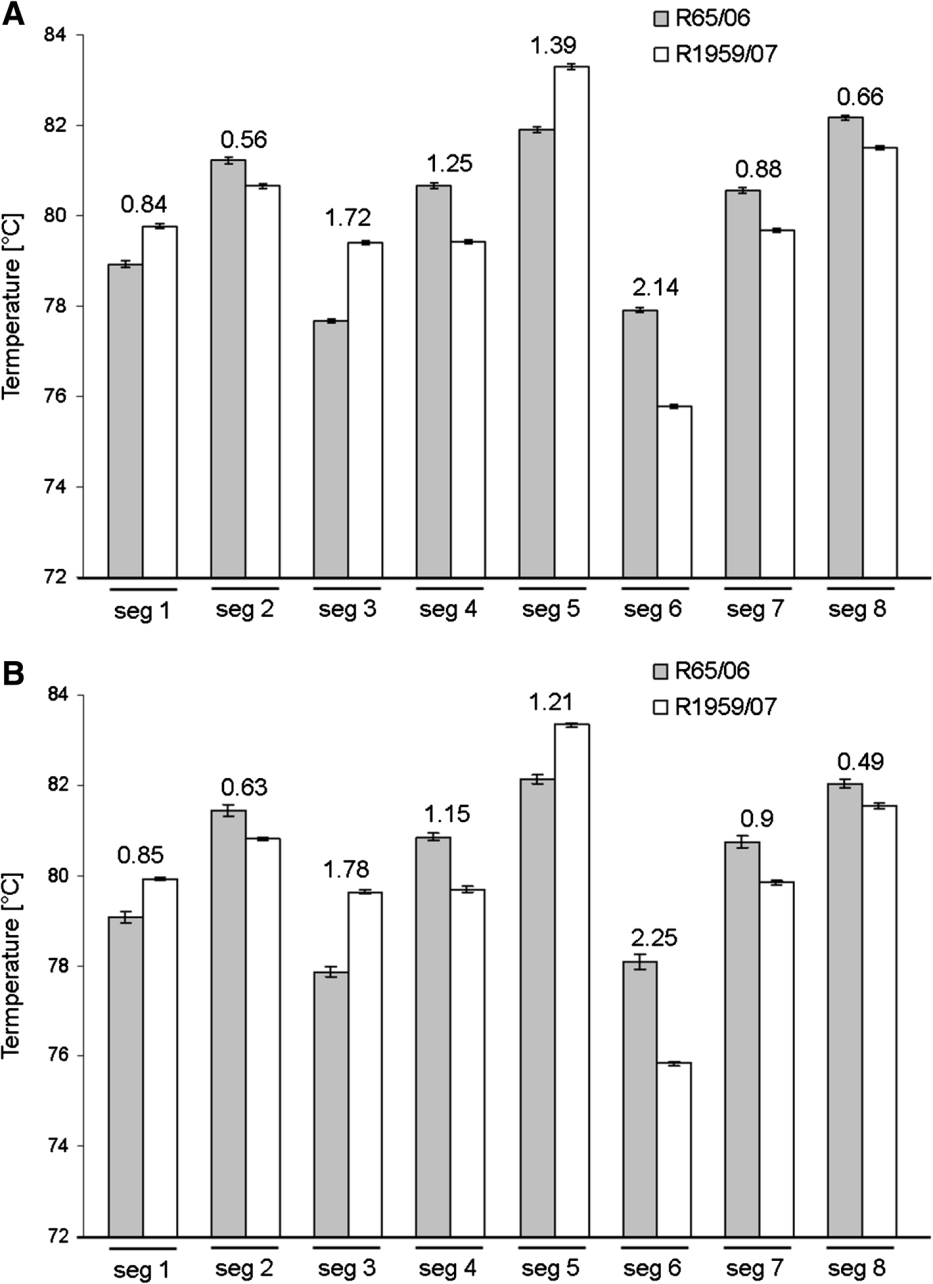
**Melting peaks detected by HRM analyses of eight segments of two closely related HPAIV strains.** The numerical difference between the melting peaks is indicated above the columns. **(A)** Melting peaks detected using the Light Cycler system. **(B)** Melting peaks detected using the Eco Cycler system. The values represent the average of the eight RNA preparations tested. The error bars indicate the standard deviation.

Figure 1 shows a comparison of the average melting peaks of the individual RNA segments of both influenza virus strains. The most distinct melting peaks were obtained for those segments with three SNPs (two cytosines and one guanine), whereas the corresponding sequence consisted of adenine and thymines, such as that obtained for segments 3 and 6 (Figure 1 and Table 1). However, as few as two SNPs within a sequence resulted in an explicit differentiation of 0.88°C and 0.9°C, e.g., as obtained for segment 7 (Figure 1 and Table 1). The smallest melting peak differences were obtained for segments 2 and 8 (values < 0.49/0.6, Figure 1), which exhibited four SNPs consisting of three cytosines/guanines and one adenine/thymine in one sequence compared with three adenines/thymines and one cytosine/guanine in the other sequence. Therefore, the SNP length is less pivotal than the SNP composition for the generation of large differences between the melting peaks of two segment sequences.

**Table 1:** Virus segment-specific PCR primers and corresponding SNPs

	**Designation**	**Sequence 5‘→3’**	**Amplicon length (bp)**	**No. of SNP**	SNPs in R65 (top row) versus R1959 (bottom row) = *
Segment 1 (PB2)	R65-PB2-1648Fw	GGTCCTGAGTCAGTGCTTG	94	4	GAAT
	R65–PB2-1742Rv	CGGTTCAAACTCCATCTTATTGT			ACGG
Segment 2 (PB1)	R65-PB1-1524Fw	ATTTGTAGCCAATTTCAGTATGGA	128	4	CAGG
	R65-PB1-1652Rv	TGAAGAGCCATCTGAGCTG			TGTA
Segment 3 (PA)	R65-PA-1547Fw	GATCCCACTTGAGGAATGATAC	78	3	TTA
	R65-PA-1625Rv	CAGTACTTTTCCCACTTGTGTG			CCG
Segment 4 (HA)	R65-HA 204Fw	ATCTAGAYGGAGTGAAGCCTC	85	3	GCC
	R65-HA 289Rv	TAAGACCATTCCGGCACATTG			ATT
Segment 5 (NP)	R65-NP-355Fw	TGGGTGAGAGAGCTGATTCTGTACG	113	3	ATT
	R65-NP-468Rv	TGGAATGCCATATCATCAGGTG			GCC
Segment 6 (NA)	R65-NA-1156Fw	GGTCAGGATATAGCGGGAG	84	3	CGC
	R65-NA-1240Rv	ATTAACTCAACCCAGAAACAAGG			TAT
Segment 7 (M)	R65-M-119Fw	TTTGCAGGAAAGAACACCGATC	93	2	CC
	R65-M-212Rv	CAAATCCCAACATCCCTTTAGTC			TT
Segment 8 (NS)	R65-NS1-224Fw	GCGAATTCTGGAGGAGGAG	112	4	CACC
	R65-NS1-336Rv	AAGGGAACCTGTCACTTTCTG			TGAT

In total, 18 different viruses were engineered by reverse genetics (two ancestor viruses, eight viruses consisting of seven segments from strain R65 and one segment from strain R1959, and eight viruses consisting of seven segments from R1959 and one segment from R65). The RNA preparations of these 18 viruses were tested on both cycler systems, and all eight segments of each virus were evaluated. All of the virus strains were correctly identified using both cycler systems (Additional file 1: Table S1).

The average melting peaks detected by the Eco cycler system did not differ by more than 0.3°C from the average melting peaks detected with the Light cycler system. However, the melting peaks detected with the Light cycler system exhibited lower standard deviations compared with those obtained with the Eco cycler system. Nevertheless, the average differences between the melting peaks of the individual influenza segments obtained with both cycler systems exhibited very similar values (Figure 1). Although the classical approach used for the verification of recombinant influenza reassortants is based on sequencing the relevant parts of the viral genome, our approach identified the reassortant gene segment composition directly through the HRM technique. A prerequisite for this method is a set of primer pairs that cover all of the SNP positions that distinguish the viral segments. Therefore, sufficient sequence information must be available before suitable primers can be designed.

In our opinion, the use of universal primers, i.e., primers that are applicable to all influenza genomes, is unfavourable because the efforts and costs associated with the design of specific primers for each tested sequence are low. In addition primer design of universal primers that generated an amplicon suitable for HRM analysis for all divergent influenza viruses would be hard to achieve.

The virus strains tested in this study were selected because they are closely related and thus difficult to differentiate. However, the applied system discriminated the sequences correctly without any problems, and the distinction of more distantly related sequences (comprising more SNP positions) should therefore be even easier using the proposed HRM analysis. Further potential applications of this technology include the screening of genetically engineered influenza viruses to determine whether reassortment or mutation has occurred and the identification of reassortant viruses from field samples.

## Conclusion

In conclusion, HRM is a valuable tool for the rapid and easy identification of reassortant influenza viruses in various settings because the total costs for genotyping by HRM are low, i.e., only a simple PCR system and a generic dye are needed.

## Methods

### Viruses, primers, and isolation of viral RNA

**Figure 2:**
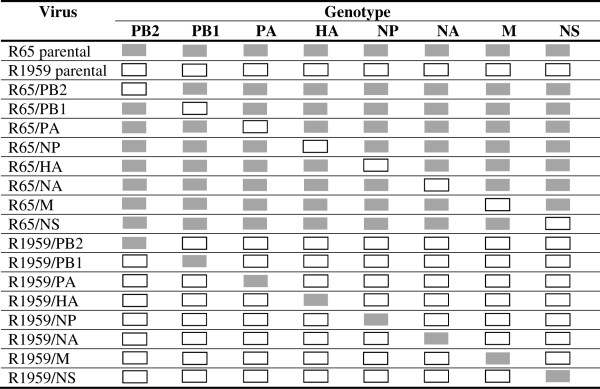
Genomic segment composition of the analysed influenza a strains.

Two highly pathogenic avian influenza viruses (HPAIV) of subtype H5N1: A/swan/Germany/R65/2006 [Gisaid: EPI103081, EPI103089, EPI103087, EPI103075, EPI103085, EPI103077, EPI103079, and EPI103083] and A/Beijing duck/Germany/R1959/2007 [EPI171617, EPI171618, EPI171619, EPI171620, EPI171621, EPI171622, EPI171623, and EPI171624] were genetically engineered (13, Eck unpublished). The reassortant viruses, which were composed of seven segments of one H5N1 strain and one segment of the sister strain, were engineered; these reassortants are called “7 + 1 reassortants”. Figure 2 summarises the unique segment composition of each of the 18 strains generated in this study. Stocks of the original wild-type viruses (R65/06 and R1959/07) were prepared using embryonated chicken eggs. The recombinant virus was rescued as described previously [15] and propagated using Madin-Darby canine kidney cells (MDCK, collection of cell lines in veterinary medicine, FLI Insel Riems, RIE1061). The viral RNA was extracted from the cell culture or allantoic fluid using the QIAamp viral RNA kit (Qiagen) according to the manufacturer’s instructions.

### Primer design

The sequence alignment of the original virus strains was used to identify the SNP positions, and specific primers were designed to cover these SNP sequences in each of the eight segments. Therefore, eight primer pairs were designed to obtain an amplicon length ranging from 78 bp to 128 bp. The SNPs consisted of at least two and at the most four nucleotides (Table 1).

### Quantitative RT-PCR

A one-step reverse transcription quantitative polymerase chain reaction (RT-qPCR) protocol was performed using the real-time ready RNA-virus-master kit (Roche Applied Science, Mannheim, Germany). The RT-qPCR assay was optimised using a total volume of 10 μl. Briefly, for each single PCR reaction, 5.05 μl of RNase-free water, 2.0 μl of 5× reaction buffer, 0.2 μl of 50× enzyme-blend, 0.5 μl of the primer mix (20 pmol/μl of both primers), and 0.25 μl of LightCycler ResoLight dye (Roche) were pooled to generate a master mix. Then, 2 μl of the RNA template was added to each reaction. The RT-qPCR reactions were performed on both an LightCycler 480 II instrument (Roche) and an Eco Real-Time PCR System (Illumina, San Diego, USA) using a single temperature profile: 8 min at 58°C (reverse transcription), 30 sec at 95°C (inactivation reverse transcriptase/activation Taq polymerase), and 45 cycles of 1 sec at 95°C (denaturation), 20 sec at 55°C (annealing), and 1 sec at 72°C (elongation). The quantification was analysed within each annealing step.

### High-resolution melting analysis

The HRM curves were obtained by incubating the PCR products at 95°C for 1 min and then subjecting them to a renaturation step of 70°C for 2 min. In the following melting step, in which the temperature was increased from 70°C to 90°C, 23 fluorescence signal acquisitions per degree centigrade were detected with the Light cycler system (ramp rate of 0.02°C/s), whereas the Eco Cycler system detected 10 signals per degree centigrade (ramp rate of 0.08°C/s). The analyses of the HRM data were performed using the Light Cycler Software (Version 1.5) and the Eco™ Software (v3.0.16.0), respectively (fluorescence signal normalisation is implemented in the software).

The individual RNA segments of the 18 virus strains (reassortants and wild type) were tested on both the Light Cycler system and the Eco Cycler system. The Cq-values determined ranged from 14.2 to 29.1, and Figure 1 shows the average value of eight replicates. The RNA (non-DNA) amplification results were verified by analysing one dataset after it was subjected to DNAse treatment (data available upon request).

Additional file 1: Table S1 presents the results of all of the analysed viral reassortants as row data, and Additional file 2: Figure S1 summarises the derivative plots for each influenza virus segment.

## Abbreviations

Cq: Quantification cycle; HPAIV: Highly pathogenic avian influenza virus; HRM: High-resolution melting; IAV: Influenza a virus; PCR: Polymerase chain reaction; RT: Reverse transcription; SNP: Single nucleotide polymorphisms; Tm: Melting temperature.

## Competing interests

The authors declare that they have no competing interests.

## Authors’ contributions

DK and BH conceived, designed, and performed the experiments, and DK, BH, and MB analysed the data. All of the authors read and approved this manuscript.

## Authors’ information

Donata Kalthoff is a veterinarian at the Institute of Diagnostic Virology at Friedrich-Loeffler-Institut in Greifswald-Insel Riems. Her research interests are focused on pathogenesis and vaccine development.

## Supplementary Material

Additional file 1: Table S1 Virus segment specific melting peak values. Click here for file

Additional file 2: Figure S1 Derivative plots representing the data for all eight influenza A virus segments. The plots were generated using the Light Cycler System. Click here for file
