# Supplementary material for: High resolution melting analysis: rapid and precise characterisation of recombinant influenza A genomes
Source: Virol J. 2013 Sep 12;10:284. doi: 10.1186/1743-422X-10-284 (PMC3847122; doi:10.1186/1743-422X-10-284)
Supplement: Additional file 1: Table S1 — Virus segment specific melting peak values. [file 1743-422X-10-284-S1.doc]

Table S1: Virus segment specific melting peaks analyzed from reverse genetically engineered viruses (ancestor viruses and 7+1 reassortant viruses) and detected by the Light Cycler system and the Eco cycler system. Sequences specific for strain R65 are illustrated by grey background color, while sequences specific for strain R1959 are indicated by a white background color. The designation of the reassortant viruses are according to Table 2.

Each column represents the composition of one newly generated recombinant virus.

LightCycler

|  | reassortant ancestor virus | |  | 7+1 reassortant viruses (seven segments from R65 and one segment from R1959) | | | | | | | |
| --- | --- | --- | --- | --- | --- | --- | --- | --- | --- | --- | --- |
|  |  |
|  | R65/06 | R1959/07 |  | R65/PB2 | R65/PB1 | R65/PA | R65/HA | R65/NP | R65/NA | R65/M | R65/NS |
| seg1/PB2 | 79.02 | 79.81 |  | 79.65 | 78.87 | 79.04 | 78.97 | 78.88 | 78.92 | 78.83 | 78.92 |
| seg2/PB1 | 81.33 | 80.59 |  | 81.20 | 80.58 | 81.37 | 81.20 | 81.17 | 81.20 | 81.16 | 81.19 |
| seg3/PA | 77.78 | 79.38 |  | 77.67 | 77.64 | 79.45 | 77.71 | 77.64 | 77.66 | 77.65 | 77.72 |
| seg4/HA | 80.74 | 79.42 |  | 80.65 | 80.63 | 80.79 | 79.41 | 80.63 | 80.64 | 80.58 | 80.72 |
| seg5/NP | 81.99 | 83.31 |  | 81.90 | 81.85 | 82.03 | 81.90 | 83.22 | 81.90 | 81.84 | 81.90 |
| seg6/NA | 78.00 | 75.74 |  | 77.89 | 77.89 | 77.99 | 77.92 | 77.87 | 75.71 | 77.84 | 77.95 |
| seg7/M | 80.69 | 79.68 |  | 80.53 | 80.51 | 80.62 | 80.54 | 80.51 | 80.53 | 79.59 | 80.58 |
| seg8/NS | 82.24 | 81.47 |  | 82.13 | 82.18 | 82.27 | 82.18 | 82.11 | 82.18 | 82.10 | 81.39 |

LightCycler

|  | reassortant ancestor virus | |  | 7+1 reassortant viruses (seven segments from R1959 and one segment from R65) | | | | | | | |
| --- | --- | --- | --- | --- | --- | --- | --- | --- | --- | --- | --- |
|  |  |
|  | R65/06 | R1959/07 |  | R1959/PB2 | R1959/PB1 | R1959/PA | R1959/HA | R1959/NP | R1959/NA | R1959/M | R1959/NS |
| seg1/PB2 | 78.91 | 79.74 |  | 78.91 | 79.74 | 79.86 | 79.74 | 79.78 | 79.74 | 79.74 | 79.84 |
| seg2/PB1 | 81.23 | 80.66 |  | 80.60 | 81.24 | 80.74 | 80.72 | 80.63 | 80.64 | 80.60 | 80.72 |
| seg3/PA | 77.77 | 79.41 |  | 79.35 | 79.38 | 77.82 | 79.46 | 79.38 | 79.37 | 79.43 | 79.47 |
| seg4/HA | 80.77 | 79.42 |  | 79.38 | 79.38 | 79.46 | 80.8 | 79.42 | 79.39 | 79.41 | 79.52 |
| seg5/NP | 82.01 | 83.29 |  | 83.23 | 83.24 | 83.39 | 83.37 | 81.93 | 83.26 | 83.25 | 83.39 |
| seg6/NA | 78.01 | 75.80 |  | 75.73 | 75.78 | 75.78 | 75.84 | 75.72 | 77.90 | 75.74 | 75.86 |
| seg7/M | 80.63 | 79.70 |  | 79.63 | 79.66 | 79.72 | 79.74 | 79.64 | 79.64 | 80.68 | 79.73 |
| seg8/NS | 82.27 | 81.48 |  | 81.51 | 81.51 | 81.60 | 81.51 | 81.44 | 81.51 | 81.51 | 82.29 |

EcoCycler

|  | reassortant ancestor virus | |  | 7+1 reassortant viruses (seven segments from R65 and one segment from R1959) | | | | | | | |
| --- | --- | --- | --- | --- | --- | --- | --- | --- | --- | --- | --- |
|  |  |
|  | R65/06 | R1959/07 |  | R65/PB2 | R65/PB1 | R65/PA | R65/HA | R65/NP | R65/NA | R65/M | R65/NS |
| seg1/PB2 | 79.1 | 79.9 |  | 79.8 | 78.9 | 79.2 | 79.2 | 79.2 | 79 | 79 | 78.9 |
| seg2/PB1 | 81.4 | 80.8 |  | 81.3 | 80.7 | 81.6 | 81.5 | 81.6 | 81.3 | 81.5 | 81.3 |
| seg3/PA | 77.9 | 79.6 |  | 77.8 | 77.7 | 79.8 | 78.1 | 77.9 | 77.8 | 77.9 | 77.8 |
| seg4/HA | 81 | 79.6 |  | 80.9 | 80.8 | 80.9 | 79.5 | 80.8 | 80.9 | 80.7 | 80.8 |
| seg5/NP | 82.1 | 83.3 |  | 82.1 | 82 | 82.3 | 82.2 | 83.4 | 82.1 | 82.2 | 82 |
| seg6/NA | 78.1 | 75.8 |  | 78 | 77.9 | 78.3 | 78.3 | 78.2 | 75.8 | 77.9 | 78 |
| seg7/M | 80.7 | 79.8 |  | 80.7 | 80.6 | 80.9 | 80.8 | 81 | 80.7 | 79.9 | 80.6 |
| seg8/NS | 82.2 | 81.4 |  | 82 | 82 | 82.1 | 81.9 | 82 | 82.1 | 81.9 | 81.3 |

EcoCycler

|  | reassortant ancestor virus | |  | 7+1 reassortant viruses (seven segments from R1959 and one segment from R65) | | | | | | | |
| --- | --- | --- | --- | --- | --- | --- | --- | --- | --- | --- | --- |
|  |  |
|  | R65/06 | R1959/07 |  | R1959/PB2 | R1959/PB1 | R1959/PA | R1959/HA | R1959/NP | R1959/NA | R1959/M | R1959/NS |
| seg1/PB2 | 79.0 | 79.9 |  | 79.0 | 79.9 | 80.0 | 79.9 | 79.9 | 79.9 | 79.9 | 79.9 |
| seg2/PB1 | 81.4 | 80.8 |  | 80.8 | 81.3 | 80.8 | 80.8 | 80.9 | 80.8 | 80.8 | 80.8 |
| seg3/PA | 77.9 | 79.6 |  | 79.6 | 79.6 | 77.9 | 79.7 | 79.7 | 79.6 | 79.7 | 79.6 |
| seg4/HA | 81.0 | 79.7 |  | 79.6 | 79.6 | 79.7 | 81.0 | 79.8 | 79.7 | 79.7 | 79.8 |
| seg5/NP | 82.1 | 83.4 |  | 83.3 | 83.3 | 83.3 | 83.4 | 82.2 | 83.3 | 83.3 | 83.4 |
| seg6/NA | 78.1 | 75.8 |  | 75.8 | 75.8 | 75.8 | 75.9 | 75.9 | 78.1 | 75.8 | 75.9 |
| seg7/M | 80.7 | 79.8 |  | 79.8 | 79.8 | 79.9 | 79.9 | 79.9 | 79.8 | 80.8 | 79.9 |
| seg8/NS | 82.2 | 81.5 |  | 81.4 | 81.5 | 81.6 | 81.6 | 81.6 | 81.5 | 81.6 | 82.3 |
