# Supplementary figures and images for: High resolution melting analysis: rapid and precise characterisation of recombinant influenza A genomes
Source: Virol J. 2013 Sep 12;10:284. doi: 10.1186/1743-422X-10-284 (PMC3847122; doi:10.1186/1743-422X-10-284)

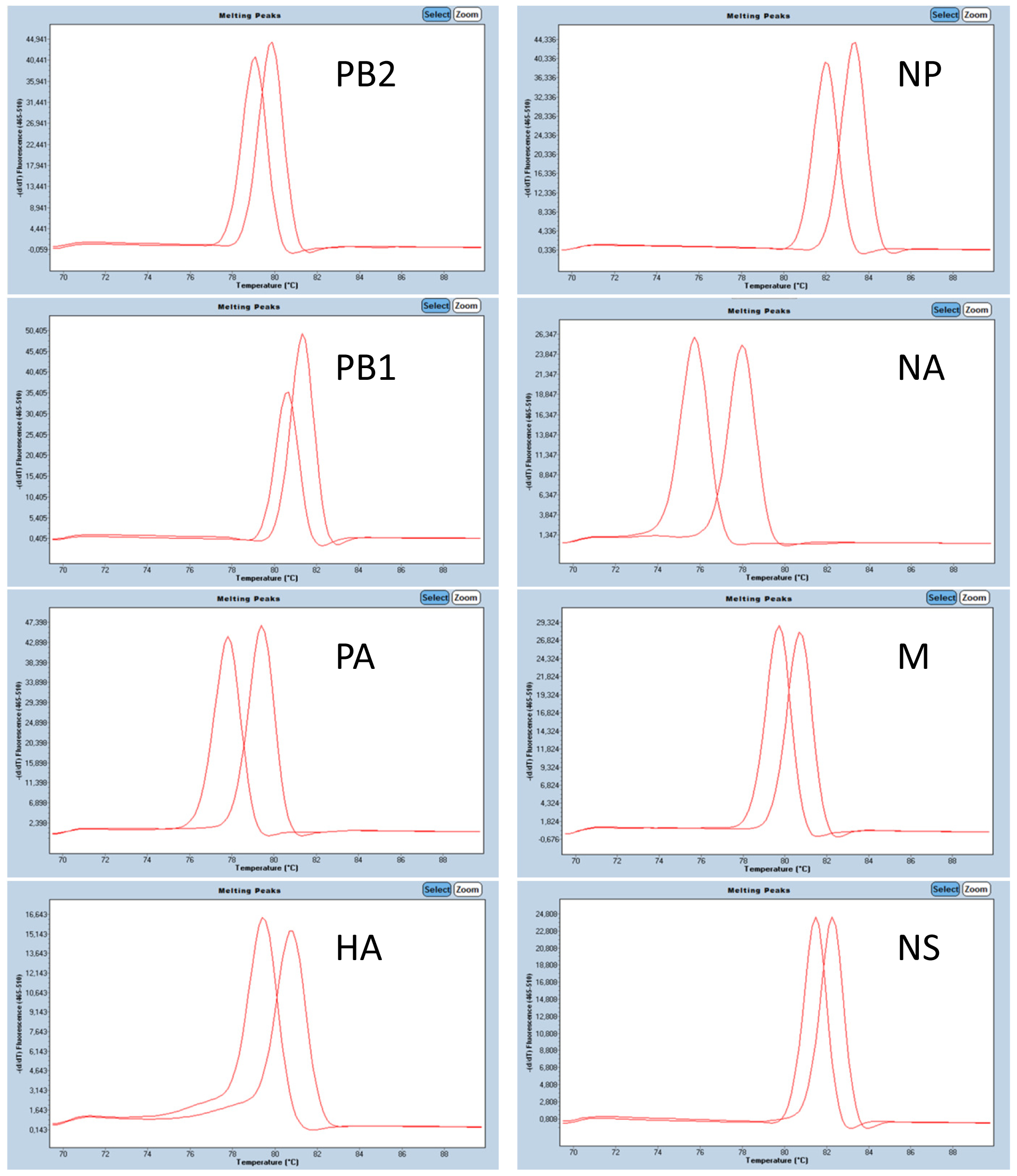

Supplement: Additional file 2: Figure S1 — Derivative plots representing the data for all eight influenza A virus segments. The plots were generated using the Light Cycler System. [file 1743-422X-10-284-S2.tiff]
